# Distinct body fat distribution and its association with metabolic syndrome in Tibetan population

**DOI:** 10.1186/s12944-025-02808-y

**Published:** 2025-11-22

**Authors:** Lin Yuan, Haijing Wang, Qingxia Huang, Tiemei Li, Bin Zhang, Huiru Tang, Youfa Wang, Wen Peng

**Affiliations:** 1 Department of Public Health, Qinghai University Medical College, Xining, Qinghai 810008 China; 2 Nutrition and Health Promotion Center, Medical College, Qinghai University, Xining, Qinghai 810008 China; 3 State Key Laboratory of Genetics and Development of Complex Phenotypes, School of Life Sciences, Human Phenome Institute, Zhangjiang Fudan International Innovation Center, Metabonomics and Systems Biology Laboratory at Shanghai International Centre for Molecular Phenomics, Zhongshan Hospital, Fudan University, Shanghai, 200433 China; 4 School of Mathematics and Statistics, Qinghai Minzu University, Xining, Qinghai 810007 China; 5 Global Health Institute, School of Public Health, Xi’an Jiaotong University, Xi’an, Shaanxi 710061 China; 6 Qinghai Provincial Key Laboratory of Prevention and Control of Glucolipid Metabolic Diseases with Traditional Chinese Medicine, Medical College, Qinghai University, Xining, Qinghai 810008 China

**Keywords:** Body fat composition, Metabolome, Lipoproteins, Metabolic syndrome, Mediation analysis

## Abstract

**Objective:**

To characterize the specific pattern of body fat distribution and its association with metabolic syndrome (MetS) among Tibetan adults, an understudied population with distinct high-altitude adaptations, and to identify potential mediating biomarkers in serum lipoprotein profiles.

**Methods:**

A total of 1480 participants from the Tibetan cohort and the NHANES were included. Principal component analysis and Mantel tests were employed to identify Tibetan-specific body fat indicators. Linear models assessed associations with metabolic syndrome (MetS), and mediation analyses evaluated the indirect effects of serum lipoproteins.

**Results:**

Tibetans showed distinct trunk and total fat mass compared to other ethnic/racial groups. Trunk fat percentage was identified as a risk factor for MetS (OR = 1.59, 95% CI: 1.27 ~ 1.91, *p* = 0.004). The triglycerides to total lipids ratio in low density lipoprotein 3 (L3TGP) and triglycerides to high density lipoprotein cholesterol ratio (TGHCR) exhibited significant mediating effect between trunk fat percentage and MetS (L3TGP:β = 1.7 × 10^− 4^g, 95% CI: 4 × 10^− 5^~3.6 × 10^− 4^, *p*<0.001;TGHCR: β = 1.8 × 10^− 4^g, 95% CI: 4 × 10^− 5^~4.6 × 10^− 4^, *p*<0.001).

**Conclusions:**

This study revealed novel evidence for distinct fat distribution in Tibetans, linked to elevated MetS risk. L3TGp and TGHCR were identified as key lipoprotein mediators, supporting the need for environmental- and ethnicity-specific indicators in metabolic risk assessment.

**Supplementary Information:**

The online version contains supplementary material available at 10.1186/s12944-025-02808-y.

## Introduction

Although body mass index (BMI) has long been regarded as a core indicator for assessing obesity and related health risks, emerging insights from in-depth obesity research and biological perspectives reveal inherent limitations in using BMI as a criterion for metabolic healthy [1]. It fails to precisely reflect the specific distribution and content of body fat, nor can it effectively determine whether excess fat has already posed health risks [2]. Studies have demonstrated that individuals with identical BMI values may exhibit markedly distinct metabolic risk due to heterogeneous fat distribution patterns (e.g., visceral fat accumulation versus subcutaneous fat deposition) [3]. Furthermore, the distribution of adipose tissue has been confirmed to significantly correlate with metabolic dysfunction, and this association may demonstrate ethnic-specific variations influenced by genetic factors and environmental adaptations [4]. Tibetans have undergone long-term high-altitude adaptation, which may have shaped unique body composition characteristics. These distinct features provide a natural model to study how environmental pressures shape metabolic disease risk through distinct body fat distribution.

The adipose tissue, an important part of body composition and also as a dynamic metabolic organ, not only participates in energy storage but also extensively regulates lipoprotein metabolism through the secretion of bioactive substances such as adiponectin and inflammatory cytokines [5]. Adipose tissue distribution exhibits a well-established association with dyslipidemia, and previous studies demonstrate that android obesity serves as an independent risk factor for lipid metabolism disorders, with high density lipoprotein cholesterol (HDL-C) levels being inversely influenced by total adiposity [6]. The association between adipose tissue distribution and lipoprotein levels exhibits heterogeneity across different populations. In males, adipose tissue distribution patterns correlate significantly with serum lipids and lipoprotein subfractions, such as the waist-to-hip ratio and waist circumference, and this effect is independent of age [7]. Among early postmenopausal women, adipose distribution parameters, specifically abdominal fat percentage or waist-to-hip ratio, constitute stronger predictors of atherogenic lipoprotein and apolipoprotein profiles than either body weight or BMI [8]. A previous study identified the association between lipoprotein profile and distinct obesity phenotypes in Tibetans [9]. However, how lipoproteins relate specifically to patterns of fat distribution in this population remains unresolved.

The distribution of adipose tissue is an important determinant of metabolic risk [10]. Metabolic syndrome (MetS) is a complex metabolic disorder, arises from complex interactions among genetic, environmental, and lifestyle factors [11]. Critically, MetS substantially increases the risk of a range of non-communicable diseases, including cardiovascular disease, non-alcoholic fatty liver disease, chronic kidney disease, and certain cancers [12]. Globally, the prevalence of MetS is estimated at approximately 25%. In China, around 454 million adults are impacted by MetS, with a national prevalence rate of 33.9% [13], while notable ethnic disparities exist. Our previous study revealed that urbanized and semi-urbanized Tibetan populations exhibit MetS prevalence rates of 30.1% in males and 32.1% in females, approaching the national average [14]. These findings suggest that, despite long-term high-altitude adaptation, Tibetan populations face a comparable burden of MetS. Yet, the mechanisms by which distinct body fat composition traits contribute to MetS remain poorly understood.

This study investigates heterogeneity in body fat distribution between Tibetans and various racial/ethnic groups using data from the National Health and Nutrition Examination Survey (NHANES). We further examined whether such heterogeneity directly or indirectly contributes to elevated MetS risk. By identifying population-specific adiposity profiles and uncovering the mediating roles of key serum lipoproteins, we provide a potential mechanistic insight into ethnic differences in metabolic health and highlight the need for precision approaches to risk assessment and prevention in diverse populations.

## Methods

### Study design and participants

This study utilized a cross-sectional design, with data from an independently established High-Altitude Multi-Ethnic Cohort, and integrated participants from the NHANES database. All data for NHANES study were acquired from the Centers for Disease Control and Prevention website (https://wwwn.cdc.gov/Nchs/Nhanes). Specifically, it included participants from the NHANES 2017–2018 survey cycle and the 2022 cross-sectional data from the High-Altitude Multi-Ethnic Cohort. To enhance the comparability of two cohorts, we minimized potential demographic confounding by matching the Tibetan and NHANES participants on age and sex at birth. The High-Altitude Multi-Ethnic Cohort was established in Golmud City, Haixi Prefecture, Qinghai Province, China. The main participants in this cohort are Tibetans, and the baseline surveys commenced in 2018, with supplementary recruitment of new participants conducted between December 2021 and May 2022 [9]. Both databases employed the same technical standard of dual-energy X-ray absorptiometry (DXA) for body composition assessment.

**Fig. 1:**
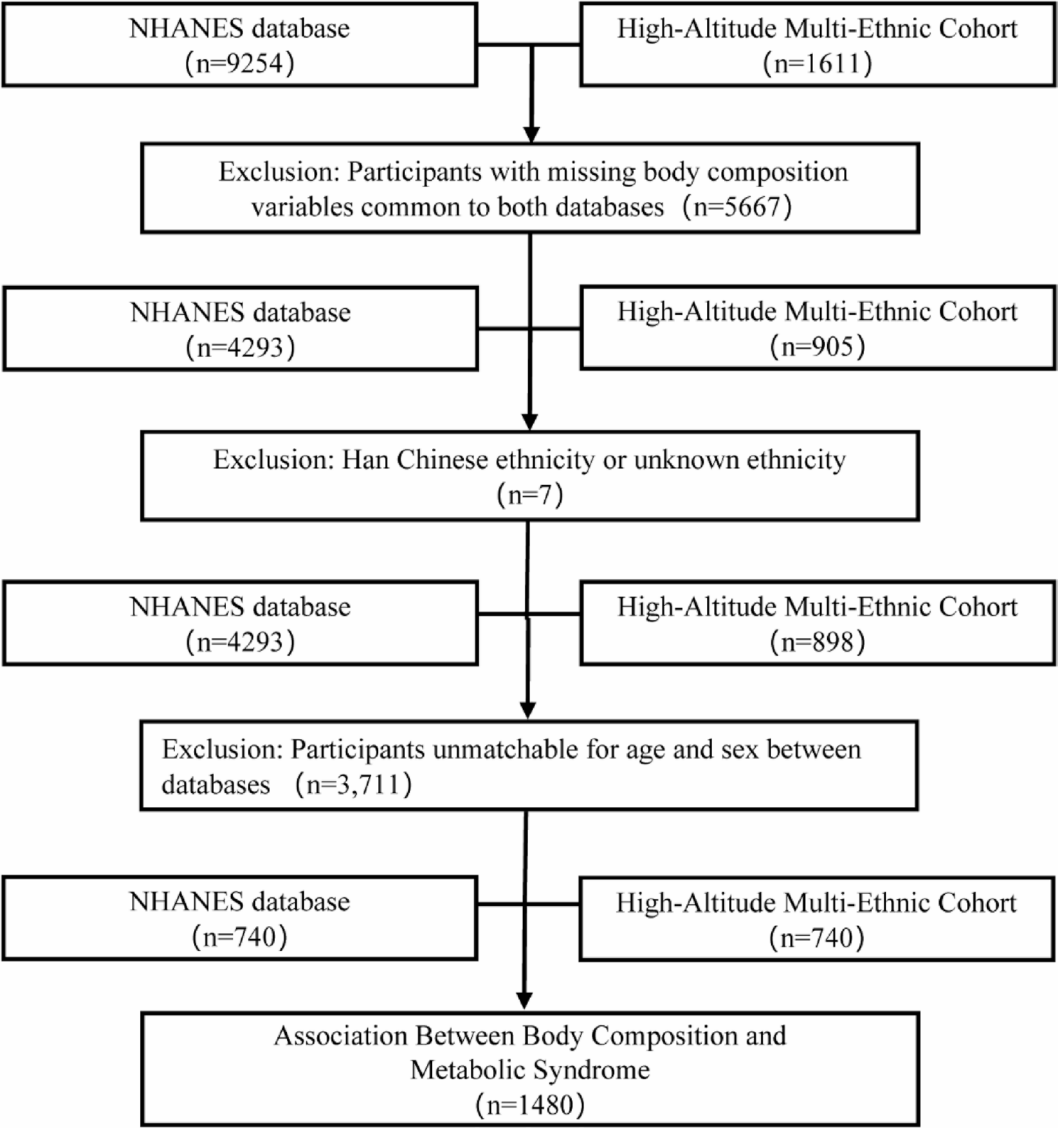
Flowchart of participant data screening

A total of 10,865 participants were initially considered. Exclusion criteria were applied as follows (Fig. 1): (1) Individuals with missing body composition data were removed from both databases (NHANES: *n* = 4,961; High-Altitude Multi-Ethnic Cohort: *n* = 706; total excluded = 5,667); (2) Individuals of Han Chinese or unidentified ethnicity were excluded from the High-Altitude Multi-Ethnic Cohort (*n* = 7). Then, 4,293 participants remained in the NHANES cohort and 898 in the High-Altitude Multi-Ethnic Cohort. Subsequently, 1:1 matching between the two databases was performed based on age and sex. Exact matching was used for sex at birth, while age was matched using a caliper of ± 1 year. This step excluded 3,711 individuals. Finally, 1,480 individuals were included for following analysis, comprising 740 Tibetan participants from the High-Altitude Multi-Ethnic Cohort and 740 matched participants from the NHANES. Individuals from the NHANES represented diverse racial and ethnic groups, including Hispanic (Mexican American and Other Hispanic), White, Black, Asian, and other racial backgrounds.

### Body composition measurements

DXA is recognized as a gold-standard method for assessing body composition [15]. Scans were performed with Hologic Apex software (version 4.0) following the manufacturer’s protocols. All measurements and quality control procedures were performed as previously reported [16]: (1) Subject preparation and positioning: Participants wore lightweight clothing with metal objects removed; subject repositioning was required after each scan. (2) Device calibration: Daily calibration of the equipment was performed. (3) Technician certification: All four operators completed unified training certified by the International Society for Clinical Densitometry (ISCD), covering the official ISCD technologist practical manual and the manufacturer-provided test protocols and operational guidelines. (4) Precision verification: Measurement precision was verified through triplicate scans (with subjects leaving the scanning table and being repositioned between each scan) of the lumbar spine and hip regions in fifteen participants; only a single scan per region was acquired during formal measurements. For this study, nine fat mass indices were included in the analysis.

### Covariates

In High-Altitude Multi-Ethnic cohort, physical examinations were conducted by trained physicians, including standardized measurements of height, weight, and waist circumference, taken while participants wore lightweight clothing. BMI was calculated as weight (kg) divided by height squared (m²). Covariates related to general demographics (age, sex, marital status), socioeconomic status (education, insurance, household income), and lifestyle behaviors (smoking, drinking, physical activity) were collected through face-to-face interviews conducted by certified investigators using structured questionnaires.

### Serum metabolome quantification

The lipoprotein subfractions in serum were performed on a 600 MHz AVANCE III NMR spectrometer equipped with a BBI probe (Bruker Biospin GmbH, Germany), following a previously established protocol [17–19]. After data cleaning and imputation of missing values, this study included a total of 333 quantifiable metabolomic parameters, comprising 129 lipoprotein subfractions, 165 lipoprotein-to-fatty acid ratio parameters, and 39 low-molecular-weight metabolites [9]. The detailed lipoprotein profile covered very-low-density lipoprotein (VLDL), intermediate-density lipoprotein (IDL), low-density lipoprotein (LDL), and high-density lipoprotein (HDL), along with their 15 subfractions (VLDL1–5, LDL1–6, HDL1–4). These lipoproteins and their subfractions were quantified for compositional parameters, including: apolipoproteins (Apo-A1, Apo-A2, Apo-B); total cholesterol (CH), free cholesterol (FC), cholesterol ester (CE); phospholipids (PL), triglycerides (TG). Additionally, functional parameters derived from the aforementioned quantitative data were also calculated, such as the cholesterol-to-triglyceride ratio (CHTGR) and lactate-to-pyruvate ratio (LactPyR).

### Laboratory and clinical measurements

Blood pressure was measured by certified staff with a Panasonic EW3106 electronic sphygmomanometer on the right upper arm after participants had rested quietly in a seated position for ≥ 5 min, two readings were averaged for analysis. Venous blood was collected after an overnight fast (≥ 8 h) and all assays were performed by the accredited laboratory of the Second People’s Hospital of Golmud.

### Definition of metabolic syndrome

Metabolic syndrome was defined in this study as the presence of at least three of the following criteria [20]: (a) Elevated WC (waist circumference): WC ≥ 90 cm for males and ≥ 80 cm for females; (b) Elevated BP (blood pressure): systolic blood pressure (SBP) ≥ 130 mmHg or diastolic blood pressure (DBP) ≥ 85 mmHg or on antihypertensive medication; (c) Elevated fasting glucose: fasting plasma glucose (FBG) ≥ 5.6 mmol/L or on medication for high blood glucose; (d) Reduced HDL-C: HDL-C < 1.03 mmol/L for males and < 1.30 mmol/L for females or on medication for reduced HDL-C; (e) Elevated TG: TG ≥ 1.7 mmol/L or on medication for elevated TG.

### Statistical analysis

Continuous variables were described using mean ± standard deviation (SD) for normally distributed data and compared via Student’s t-test or analysis of variance (ANOVA), while non-normally distributed data were reported as median (interquartile range) and analyzed using Wilcoxon rank-sum or Kruskal-Wallis tests. Categorical variables were summarized as frequencies (proportions) and assessed with chi-square tests.

Principal component analysis (PCA) was employed to explore the distribution patterns of body fat-related variables across ethnic/racial groups following dimensionality reduction, selecting adiposity indicators that were significantly represented in the top 10 loading variables of both PC1 and PC2. Ethnic characteristics were analyzed using Mantel tests and Spearman correlations to dual-validate the robustness of the PCA-selected indicators.

Additionally, linear regression models adjusted for age, sex, marital status, education level, health insurance coverage, household income, smoking status, alcohol consumption, and physical activity were used to assess differences in two key adiposity indices between Tibetans and other ethnic/racial groups. To eliminate confounding by body weight, subsequent analyses utilized the ratio of key adiposity indices to body weight. The trunk fat percentage and total fat percentage were defined as trunk fat mass/weight and total fat mass/weight, respectively.

Given the high dimensionality and strong collinearity of serum lipoprotein data, we integrated linear regression, logistic regression, and least absolute shrinkage and selection operator (LASSO) regression (via the R glmnet package) for variable selection. Specifically, we first identified metabolites significantly associated with trunk fat percentage using linear regression, and with MetS using logistic regression. To account for multiple testing across the numerous metabolites, the Benjamini-Hochberg (BH) method was applied to control the false discovery rate (FDR). Only variables with an FDR-adjusted *p* < 0.05 in both regression analyses were considered significant and retained for the next step. Secondly, LASSO regression was performed on metabolites that showed significance (FDR-adjusted *p* < 0.05) in both of the initial regression analyses. Finally, mediation analysis was conducted using the R mediation package, with bootstrap resampling (1,000 iterations) to estimate confidence intervals for mediation effects, thereby elucidating the intermediary roles of serum lipoproteins.

Statistical significance was defined as a two-tailed *p* < 0.05, and all analyses were performed in R version 4.4.1.

## Results

### Identification of key body fat indicators among ethnic/racial groups

To minimizing potential confounding effects from demographic disparities and enhancing the comparability of two cohorts, age and sex matching was performed between Tibetan participants and other ethnic/racial groups from the NHANES database. The median age across all groups ranged from 42 to 46 years, with no significant differences in age and sex, while significant difference was observed in marital status and BMI (Table 1).

**Table 1:** Demographic characteristics among Tibetan and other ethnical/racial groups

	Mexican American	Non-Hispanic (Asian)	Non-Hispanic (Black)	Non-Hispanic (Other)	Non-Hispanic (White)	Other Hispanic	Tibetan	*p*
	*N* = 98	*N* = 151	*N* = 132	*N* = 48	*N* = 231	*N* = 80	*N* = 740	
Age	42.00 (33.00–51.00)	46.00 (37.00–52.00)	45.00 (36.00–52.00)	45.50 (37.00–52.50.00.50)	43.00 (33.00–51.00)	45.50 (37.00–52.50.00.50)	44.00 (35.00–52.00)	0.120
Sex (%)								0.160
Men	44 (44.90)	62 (41.10)	66 (50.00)	27 (56.30)	96 (41.60)	27 (33.80)	322 (43.50)	
Women	54 (55.10)	89 (58.90)	66 (50.00)	21 (43.80)	135 (58.40)	53 (66.30)	418 (56.50)	
Marital status (%)								< 0.001
Unmarried	17 (17.70)	11 (7.30)	46 (35.40)	11 (22.90)	44 (19.50)	9 (11.40)	82 (11.20)	
Marital	63 (65.60)	126 (83.40)	61 (46.90)	25 (52.10)	144 (63.70)	53 (67.10)	620 (84.70)	
Others	16(16.70)	14(9.30)	23(17.70)	12 (25.00)	38(16.80)	17(21.50)	30(4.10)	
BMI (kg/m ^2^)	28.50(26.00–32.50.00.50)	25.50(22.80–28.60)	28.10(24.60–36.20)	28.40(24.30–32.60)	27.40(23.80–32.90)	28.20(25.00–31.60.00.60)	26.50(23.00–29.70.00.70)	< 0.001

**Fig. 2:**
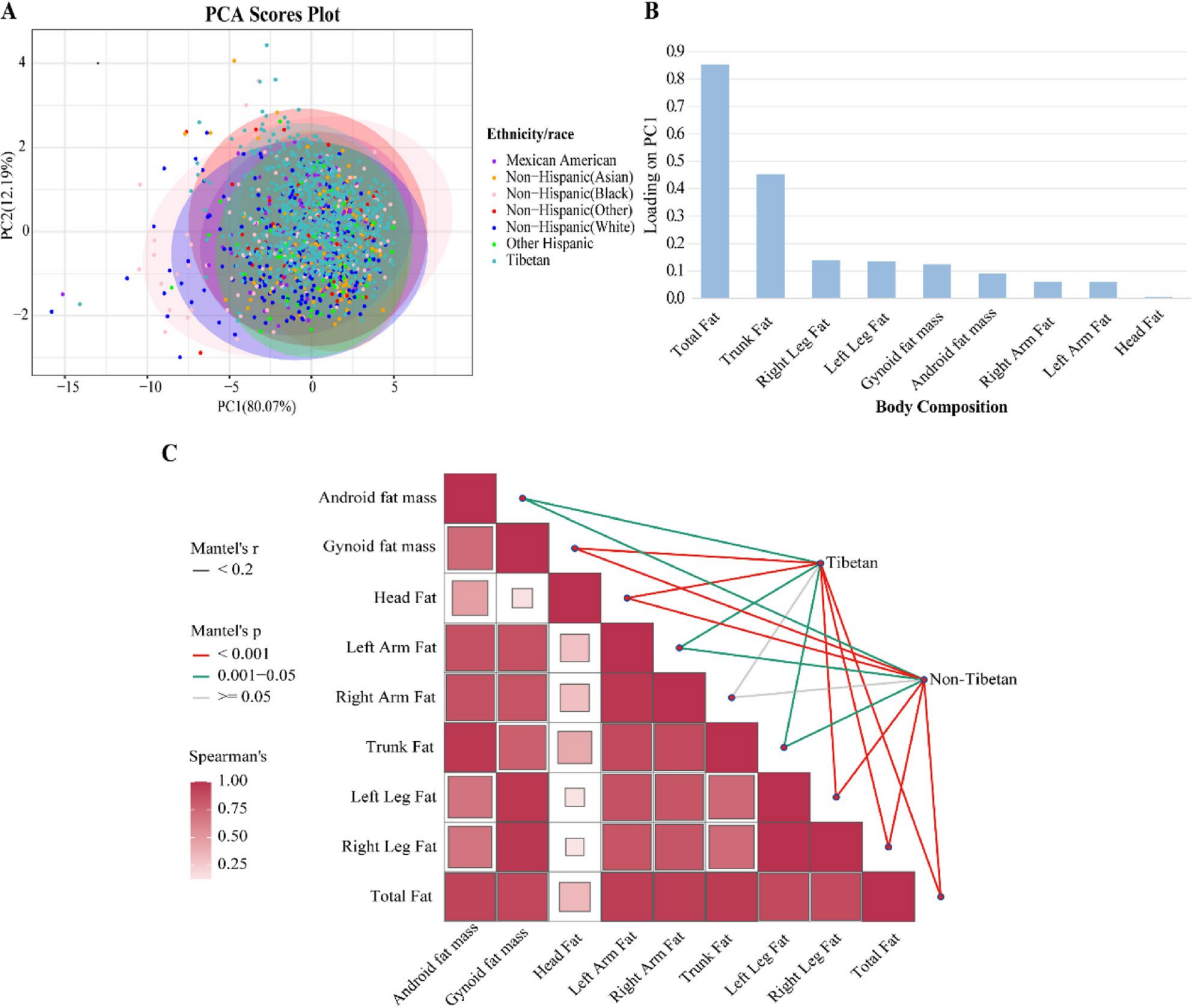
Key body fat indicators were identified across ethnic/racial groups using multimodal approaches.**A** Principal component analysis (PCA) visualizes body fat indicators, with distinct colors representing different ethnic/racial groups. **B** Loading values of variables in Principal Component 1 (PC1) are ranked to highlight their contributions. **C** Mantel test for nine body fat indicators among different ethnic/racial groups

Furthermore, principal component analysis (PCA) was performed on nine body fat mass indicators common to both databases following normalization. The PCA plot demonstrated that the first principal component (PC1) explained 80.07% of the total variance, suggesting a potential separation from non-Hispanic White and Black participants (Fig. 2A). Significant differences in both PC1 and PC2 scores were also observed among ethnic/racial groups, collectively demonstrating ethnic divergence in body fat distribution. Specifically, as evidenced in box plots, the Tibetans exhibits significant differences from most Non-Hispanic groups (such as Non-Hispanic Asian, Non-Hispanic Black, and Non-Hispanic White) on PC1. On PC2, significant differences are observed between the Tibetan population and the aforementioned groups except for Non-Hispanic Other (Figure S1). Analysis of PC1 loading scores identified total fat mass and trunk fat mass as the top two contributors, underscoring their dominant influence on this principal component (Fig. 2B). To validate their discriminative capacity, mantel tests confirmed statistically significant differences in total fat mass and trunk fat mass across ethnic/racial groups, further supporting their role as dominant indicators of ethnic/racial-specific body fat distribution (Fig. 2C, Table S1).

### Differences in key body fat indicators among ethnic/racial groups

To further control the potential confounding effects of BMI and marital status, linear regression models were applied. Using Tibetans as the reference group, Mexican Americans exhibited a higher trunk fat mass 0.31 g (β = 0.31, 95% CI: 0.10 ~ 0.52, *p* = 0.004) and total fat mass 0.28 g (β = 0.28, 95% CI: 0.07 ~ 0.49, *p* = 0.009). A similar trend was observed among White and Black individuals. Conversely, Non-Hispanic Asians exhibited significantly lower trunk fat mass (β = −0.22, 95% CI: −0.39~ −0.05, *p* = 0.012) and total fat mass (β = −0.18, 95% CI: −0.35~ −0.01, *p* = 0.041) compared to Tibetans (Table 2). The results demonstrated significant differences of trunk and total fat between Tibetans and other major ethnic groups, including Mexican Americans, non-Hispanic Asians, non-Hispanic Blacks, and non-Hispanic Whites.

**Table 2:** The Trunk/Total fat mass (g) distribution among Tibetan and other ethnical/racial groups

	Trunk fat(g)		Total fat(g)	
	β(95%CI)	*p*	β(95%CI)	*p*
Tibetan	Reference		Reference	
Mexican American				
Model1	0.28 (0.07, 0.48)	0.010	0.25 (0.04, 0.45)	0.021
Model2	0.31 (0.10, 0.52)	0.004	0.28 (0.07, 0.49)	0.009
Non-Hispanic (Asian)				
Model1	−0.20 (−0.37, −0.03)	0.024	−0.16 (−0.33, 0.02)	0.076
Model2	−0.22 (−0.39, −0.05)	0.012	−0.18 (−0.35, −0.01)	0.041
Non-Hispanic (Black)				
Model1	0.17 (−0.01, 0.36)	0.066	0.39 (0.21, 0.58)	< 0.001
Model2	0.30 (0.11, 0.49)	0.002	0.52 (0.34, 0.71)	< 0.001
Non-Hispanic (Other)				
Model1	0.15 (−0.14, 0.44)	0.300	0.20 (−0.09, 0.49)	0.178
Model2	0.22 (−0.07, 0.51)	0.138	0.27 (−0.02, 0.56)	0.067
Non-Hispanic (White)				
Model1	0.30 (0.15, 0.45)	< 0.001	0.38 (0.24, 0.53)	< 0.001
Model2	0.34 (0.19, 0.49)	< 0.001	0.42 (0.28, 0.57)	< 0.001
Other Hispanic				
Model1	0.03 (−0.20, 0.25)	0.832	0.08 (−0.15, 0.31)	0.500
Model2	0.02 (−0.21, 0.25)	0.876	0.07 (−0.16, 0.30)	0.558

Tibetans as a reference group

Model 1 was unadjusted model

Model2 was adjusted for marital status and BMI

### Associations of key body fat percentage with MetS and its components in the Tibetan population

The observed disparities in body fat distribution between Tibetans and other ethnic/racial groups underscore the importance of trunk fat mass and total fat mass as key indicators, highlighting their potential health implications and prompting further scientific investigation. Among these 740 Tibetan participants, 162 (21.9%) had MetS, and significant differences were observed in sex, age, marital status, income level, smoking behavior, and BMI when compared with those without MetS. Specifically, within the Tibetan cohort, the MetS group displayed significantly higher median trunk fat, total fat distribution, and BMI than the healthy participants (Table S2).

To address potential confounding effects of body weight on key adiposity indicators, trunk fat percentage and total fat percentage were utilized in subsequent analyses. In the fully adjusted model (adjusted for age, sex, marital status, education, insurance, household income, smoking, drinking, physical activity, and BMI), each one unit increase in trunk fat percentage was associated with significantly higher risks of MetS (OR = 1.59, 95% CI: 1.27 ~ 1.91, *p* = 0.004), Elevated BP (OR = 1.53, 95% CI: 1.21 ~ 1.85, *p* = 0.011), Reduced HDL-C (OR = 1.61, 95% CI: 1.22 ~ 2.00, *p* = 0.017), and Elevated WC (OR = 2.28, 95% CI: 1.83 ~ 2.73, *p*<0.001). In contrast, total fat percentage showed no significant associations with these outcomes except for Elevated WC (OR = 2.16, 95% CI: 1.62 ~ 2.70, *p* = 0.005). After BMI adjustment, trunk fat percentage shows broader metabolic implications than total fat percentage (Table 3). These findings underscore the pivotal role of trunk fat percentage in the development of metabolic disorders.

**Table 3:** Associations of trunk/total fat mass percentage with metabolic syndrome and its components in Tibetan

	Trunk fat/Weight		Total fat/Weight	
	OR(95%CI)	*p*	OR(95%CI)	*p*
Metabolic syndrome				
Model1	1.83 (1.63, 2.03)	**< 0.001**	1.25 (1.07, 1.43)	**0.018**
Model2	2.34 (2.10, 2.58)	**< 0.001**	2.22 (1.93, 2.51)	**< 0.001**
Model3	2.42 (2.16, 2.68)	**< 0.001**	2.25 (1.95, 2.55)	**< 0.001**
Model4	1.59 (1.27, 1.91)	**0.004**	1.11 (0.72, 1.50)	0.605
Elevated BP				
Model1	1.79 (1.62, 1.96)	**< 0.001**	1.41 (1.25, 1.57)	**< 0.001**
Model2	1.79 (1.59, 1.99)	**< 0.001**	1.93 (1.68, 2.18)	**< 0.001**
Model3	1.84 (1.63, 2.05)	**< 0.001**	1.98 (1.72, 2.24)	**< 0.001**
Model4	1.53 (1.21, 1.85)	**0.011**	1.26 (0.85, 1.67)	0.268
Elevated fasting glucose				
Model1	1.51 (1.25, 1.77)	**0.002**	1.15 (0.90, 1.40)	0.266
Model2	1.46 (1.16, 1.76)	**0.014**	1.27 (0.90, 1.64)	0.198
Model3	1.49 (1.17, 1.81)	**0.015**	1.30 (0.91, 1.69)	0.180
Model4	1.04 (0.63, 1.45)	0.859	0.67 (0.14, 1.20)	0.131
Reduced HDL-C				
Model1	1.36 (1.11, 1.61)	**0.015**	1.00 (0.76, 1.24)	0.990
Model2	1.63 (1.34, 1.92)	**0.001**	1.60 (1.24, 1.96)	**0.010**
Model3	1.72 (1.41, 2.03)	**< 0.001**	1.69 (1.32, 2.06)	**0.006**
Model4	1.61 (1.22, 2.00)	**0.017**	1.43 (0.95, 1.91)	0.143
Elevated TG				
Model1	1.34 (1.07, 1.61)	**0.037**	0.86 (0.60, 1.12)	0.242
Model2	1.89 (1.56, 2.22)	**< 0.001**	1.63 (1.23, 2.03)	**0.016**
Model3	2.00 (1.65, 2.35)	**< 0.001**	1.74 (1.32, 2.16)	**0.009**
Model4	1.33 (0.88, 1.78)	0.219	0.81 (0.24, 1.38)	0.485
Elevated WC				
Model1	6.46 (6.18, 6.74)	**< 0.001**	3.59 (3.38, 3.80)	**< 0.001**
Model2	8.15 (7.82, 8.48)	**< 0.001**	12.73 (12.32, 13.14)	**< 0.001**
Model3	8.68 (8.33, 9.03)	**< 0.001**	14.03 (13.59, 14.47)	**< 0.001**
Model4	2.28 (1.83, 2.73)	**< 0.001**	2.16 (1.62, 2.70)	**0.005**

Model 1 was unadjusted model

Model 2 was adjusted for age and sex

Model 3 further adjusted for marital status, education, insurance, household income, smoking, drinking and physical activity

Model 4 further adjusted for BMI

### Mediating role of lipoproteins in the link between body fat percentage and MetS

**Fig. 3:**
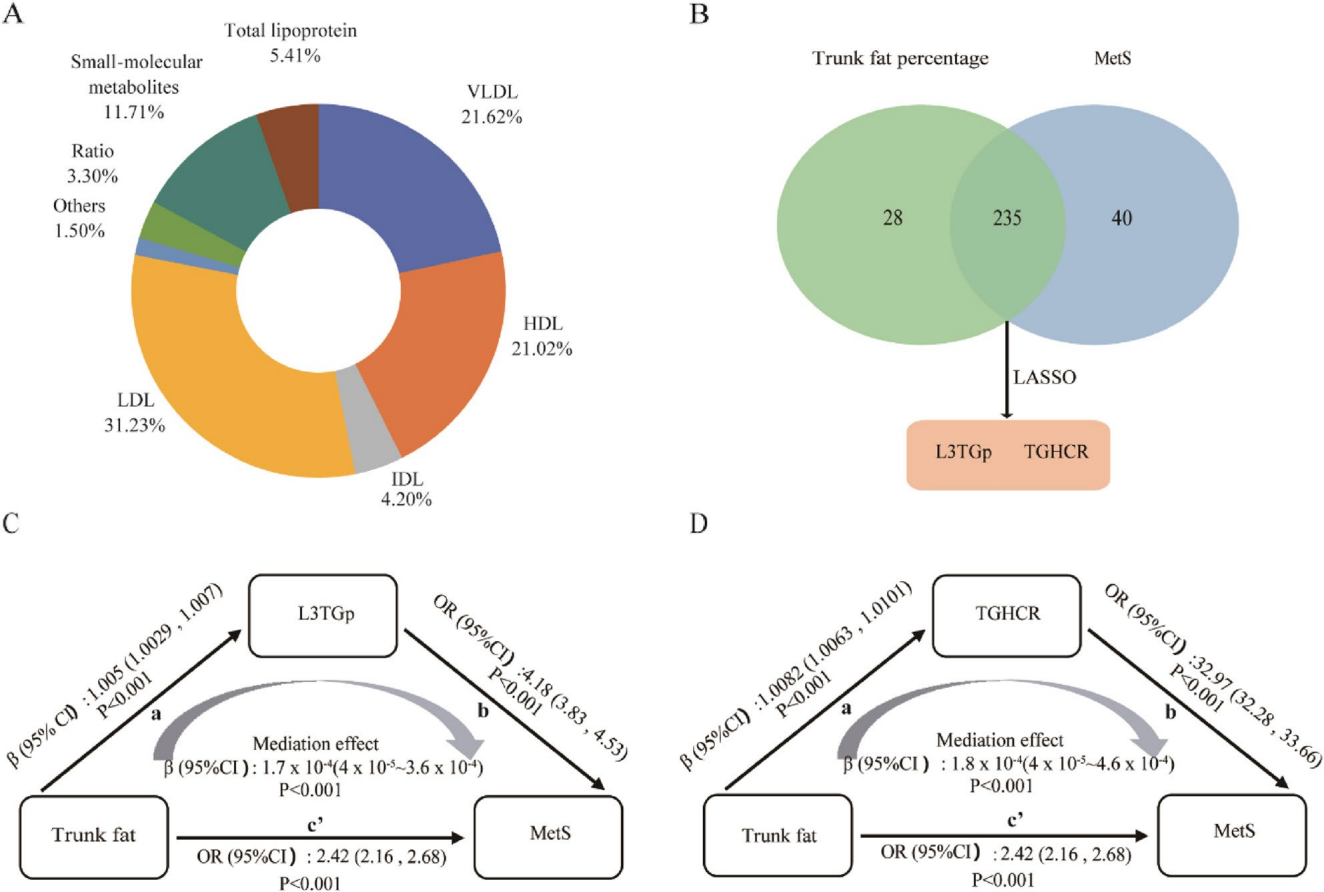
Screening of lipoproteins and its mediation effect. **A** Distribution of 333 lipid metabolism categories. **B** Results of LASSO screening for serum lipoproteins. **C** Mediation effect of L3TGp in the association between trunk fat percentage and metabolic syndrome risk. **D** Mediation effect of TGHCR in the association between trunk fat percentage and metabolic syndrome risk. ACME denotes the average causal mediation effect, and ADE represents the average direct effect. Model included adjustments for age, sex, marital status, education, insurance, household income, smoking, drinking, physical activity. The depicted paths correspond to the coefficients estimated in the mediation model: path a (Trunk fat→L3TGp/Trunk fat→TGHCR), path b (L3TGp→MetS/TGHCR→MetS), and the direct effect path c’ (Trunk fat→MetS)

To further investigate whether lipoproteins mediate the effects of trunk fat percentage on MetS, 333 quantifiable parameters such as lipoprotein subfractions and small molecular weight metabolites were included. The top three lipoprotein contributors were LDL (31.23%), VLDL (21.62%), and HDL (21.02%) subfractions and their components (Fig. 3A). Firstly, 263 metabolites significantly associated with trunk fat percentage were screened (FDR-adjusted *p* < 0.05; Table S3), and 275 metabolites significantly associated with MetS (FDR-adjusted *p* < 0.05; Table S4). Then, 235 metabolites were found to be associated with both the trunk fat percentage and MetS. Finally, two metabolites were significantly associated with MetS: triglyceride to HDL-cholesterol ratio (TGHCR) and the proportion of triglyceride to total lipid in subclass 3 of low-density lipoprotein (LDL3, L3TGp) (*p* < 0.05; Fig. 3B, Table S5). Subsequently, the mediating effects of these two metabolic markers were further investigated.

Mediation analyses showed the effect of the trunk fat percentage on MetS through metabolites, TGHCR and L3TGp, respectively. The trunk fat percentage demonstrated clear associations with MetS, with both significant mediation effects (path ab) and direct effects (path c’). Specifically, both L3TGp (β = 1.7 × 10^− 4^g, 95% CI: 4 × 10^− 5^~3.6 × 10^− 4^, *p*<0.001) and TGHCR (β = 1.8 × 10^− 4^ g, 95% CI: 4 × 10^− 5^~4.6 × 10^− 4^, *p*<0.001) demonstrated statistically significant mediating effects after adjustment for potential confounders(Fig. 3C and D). These findings suggest that the mediating effects of specific metabolites modulate the association between trunk fat and MetS.

## Discussion

This study demonstrates distinct patterns of fat distribution across racial/ethnic groups, with Tibetan populations exhibiting significant different trunk fat and total fat mass distributions compared to major racial/ethnic groups represented in the NHANES database. Furthermore, the trunk fat percentage emerged as independent risk factors for MetS within the Tibetan population. A key finding was the identification of two specific serum lipoproteins, L3TGp and TGHCR, as potential mediators in the association between trunk fat percentage and MetS. Together, these results highlight the need for ethnicity-tailored obesity metrics and support the integration of lipoprotein profiling into metabolic risk assessments.

Our findings replicate previously reported racial/ethnic differences in body fat distribution [21, 22], demonstrating that Tibetan populations exhibit a distinct adiposity pattern characterized by significantly lower trunk and total fat mass compared to Mexican-American, Black, and White individuals, yet higher levels than those observed in Asian participants within the NHANES database. Previous studies have shown that, at same levels of BMI and waist circumference, White individuals tend to have a higher visceral fat area compared to Black and Hispanic populations [23]. Chinese individuals exhibited the lowest total body fat mass yet the highest relative central distribution of whole-body and abdominal adiposity compared with Non-Hispanic Whites, Non-Hispanic Blacks, and Mexican Americans [24]. The higher total fat and trunk fat mass observed in Tibetans compared to Asian population might be related to the adaptation to hypoxic stress, and the specific fat distribution may favor subcutaneous storage and improved metabolic efficiency [16]. Long-term exposure to a hypobaric hypoxic environment at high altitudes can lead to alterations in body fat deposition sites and distribution patterns [25]. The high-altitude environment, characterized by hypobaric hypoxia, induces metabolic dysregulation, elevating oxidative stress and increasing energy demands for thermogenesis and oxygen transport [26]. In this context, subcutaneous fat may maintain oxygen supply through enhanced angiogenesis, thus reducing hypoxic damage, while its enhanced glycolytic capacity may improve hypoxia tolerance [27]. These mechanisms are consistent with our findings, suggesting an optimization of fat distribution driven by natural selection pressures to enhance the adaptation in the high-altitude environment.

The contribution of fat mass in specific regions varies in its implication for healthy. In adolescents, trunk fat has been associated with increased clustered cardiometabolic risk [28]. Among adults, trunk fat is positively correlated with elevated blood pressure, and total fat mass/height^2^ was linked to a higher risk of MetS, particularly when exceeding 26.9 kg/m^2^ [29]. Furthermore, higher percentage of total and trunk fat mass have been significantly associated with increased cardiovascular diseases mortality in NHANES based study [30]. While, trunk fat, particularly visceral fat, is more predictive of cardiometabolic risk than total fat mass [31]. Excess trunk fat, particularly visceral adipose tissue deposition, may leads to adipocyte hypertrophy, excessive extracellular matrix accumulation causing tissue fibrosis, infiltration of pro-inflammatory immune cells, and reduced protective adipokine secretion [32]. Notably, only trunk fat mass percentage was found to be significantly associated with MetS in the Tibetan population, reinforcing its role as a greater predictive power for adverse metabolic outcomes.

Trunk fat accumulation is independently associated with elevated TG, reduced HDL, and atherogenic dyslipidemia (increased total cholesterol to HDL ratio), which is consistent with the diagnostic criteria for metabolic syndrome [33]. These observations provide a compelling rationale to explore the underlying mechanisms. Here, both L3TGp and TGHCR were identified as significant mediators in the Tibetan population. The TGHCR (also known as TG/HDL-C) has emerged as a promising novel risk marker for predicting MetS [34–36]. These results highlight the critical role of relative triglyceride enrichment in the development of MetS and further support TGHCR as a robust predictor of MetS risk in this specific population [37, 38], suggesting its broader utility in metabolic risk assessment.

L3TGp, defined as the proportion of triglyceride content relative to total lipid content within LDL3 particles, was found to be significantly higher in older than in young adults [39]. LDL3 (medium-sized low-density lipoprotein particles), a subclass of LDL, has a density range of 1.034–1.037 kg/L [40]. Elevated levels of LDL3 are closely linked to increased risks of coronary heart disease, coronary atherosclerosis, and stroke [41, 42]. Excess visceral adipose tissue may accelerate lipolysis, leading to the release of large amounts of free fatty acids that are transported directly to the liver via the portal vein [43]. This process may contribute to increased hepatic synthesis and secretion of very-low-density lipoprotein, which in turn elevate circulating levels of L3TGp [44]. However, the underlying biological mechanisms require further investigation.

Here, we first examined differences in fat distribution between Tibetans, an ethnic group adapted to high-altitude environments, and other ethnically/racially groups. Furthermore, the core mediating role of specific serum lipoproteins in the association between body fat and MetS was identified in Tibetans. These findings underscore the importance of population-specific assessment tools in obesity research. They reveal distinct fat-distribution patterns among Tibetans and suggest that lipoprotein-mediated metabolic pathways may link adiposity to disease.

However, the cross-sectional design of this study limits the ability to draw causal inferences between fat distribution, lipoprotein profiles, and MetS. Although we enhanced the comparability of two cohorts by matching Tibetan and NHANES participants on age and sex, and further adjusted for potential confounders in subsequent models, unmeasured factors may still contribute to the observed associations. In addition, while both cohorts used the same DXA equipment and standardized protocols, scans were performed by different operators, introducing potential heterogeneity in body composition measurements. Finally, the generalizability of the findings is limited due to the unique environmental and genetic background of the Tibetan population.

## Conclusion

This study provides novel evidence that Tibetan populations exhibit distinct patterns of fat distribution compared to major racial/ethnic groups. The trunk fat was identified as independent risk factor for MetS, with specific serum lipoproteins, L3TGp and TGHCR, may serve as potential mediators. These findings underscore the critical need to move toward precision metabolic risk assessment that reflects the unique physiological adaptations and environmental exposures of diverse populations. To this end, incorporating ethnicity-specific indicator could help develop more equitable and effective strategies for the identification and prevention of obesity related metabolic disorders.

## Supplementary Information

Supplementary Material 1

## Data Availability

Some or all datasets generated during and/or analyzed during the current study are not publicly available but are available from the corresponding author on reasonable request.

## Abbreviations

BMI: Body mass index
HDL-C: High density lipoprotein cholesterol
MetS: Metabolic syndrome
NHANES: National health and nutrition examination survey
DXA: Dual-energy X-ray absorptiometry
ISCD: International society for clinical densitometry
VLDL: Very low-density lipoprotein
IDL: Intermediate-density lipoprotein
LDL: Low-density lipoprotein
WC: Waist circumference
HDL: High-density lipoprotein
CH: Total cholesterol
FC: Free cholesterol
CE: Cholesterol ester
PL: Phospholipids
TG: Triglycerides
CHTGR: Cholesterol-to-triglyceride ratio
LactPyR: Lactate-to-pyruvate ratio
BP: Blood pressure
SBP: Systolic blood pressure
DBP: Diastolic blood pressure
FBG: Fasting plasma glucose
SD: Standard deviation
ANOVA: Analysis of variance
PCA: Principal component analysis
BH: Benjamini-Hochberg method
FDR: False discovery rate
LASSO: Least absolute shrinkage and selection operator
CI: Confidence interval
OR: Odds ratio
LDL3: Subclass 3 of low-density lipoprotein
TGHCR: Triglyceride to HDL-cholesterol ratio
L3TGp: Proportion of triglyceride to total lipid in LDL3
